# Tuberculous peritonitis in a German patient with primary biliary cirrhosis: a case report

**DOI:** 10.1186/1752-1947-2-32

**Published:** 2008-01-31

**Authors:** Yilin Vogel, Jan C Bous, Guido Winnekendonk, Bernhard F Henning

**Affiliations:** 1 Department of Internal Medicine, Gastroenterology Unit, Marienhospital, Ruhr University, Herne, Germany; 2 Department of Radiology, Marienhospital, Ruhr University, Herne, Germany

## Abstract

**Background:**

The number of cases of tuberculosis as a complication in people with immunodeficiency, people on immunosuppressive therapy and among the immigrant population is increasing in Germany. However, tuberculous peritonitis rarely occurs without these risks, particularly in Germans. The incidence of tuberculous peritonitis in Germany is very low; tuberculosis of the intestinal tract was found in approximately 0.8 % of tuberculosis cases in 2004. The diagnosis of tuberculous peritonitis is often delayed on account of non-specific clinical symptoms. The absence of specific biological markers, long incubation times for cultures and non-specific radiographic or ultrasonographic signs increase the morbidity associated with this treatable condition.

**Case presentation:**

We report a case of tuberculous peritonitis in a 73-year-old female German patient. Her medical history revealed primary biliary cirrhosis (PBC) since 1992. On admission, she complained of abdominal pain, vomiting, ascites and peripheral edema. The patient has been in a seriously reduced general condition and had fever up to 39.6°C. A few weeks earlier, the patient was in another hospital with the same complaint. Inflammatory parameters were elevated, but the procalcitonin level was normal. Blood culture was always negative, as was the tuberculin test. Ultrasonography of the abdomen showed massive ascites with multiple septa. The patient underwent a computed tomography (CT) scan of the abdomen which showed a thickened intestinal wall in the sigmoid colon and a pronounced enhancement of the peritoneum. Computed tomography scans of the lung showed only slight bilateral pleural effusion. Because of the anaesthetic and bleeding risk due to thrombocytopenia, laparoscopy was not immediately undertaken. The culture from ascites was positive for *M*.*tuberculosis *after three weeks.

**Conclusion:**

In primary biliary cirrhosis patients with non-specific clinical symptoms, such as vomiting, abdominal pain, ascites, weight loss, and fever, tuberculous peritonitis must be considered in the initial differential diagnosis, although these symptoms may be attributed to cirrhosis of the liver with spontaneous bacterial peritonitis. Ultrasonographic and CT scab findings are not specific for tuberculous peritonitis, but an awareness of the ultrasonographic features and the features of the CT scan may help in the diagnosis of tuberculous peritonitis and avoid clinical mismanagement.

## Background

In industrialised countries, tuberculosis increasingly occurs in the immigrant population and in patients with acquired immune deficiency syndrome (AIDS) and those on immunosuppressive therapy. Tuberculosis of the intestinal tract ranked 8^th ^of all forms of tuberculosis (0.8%) in 2004 in Germany, after pulmonary forms (79.6%), extrathoracic lymph nodes (7%), pleura (3.6%), genitourinary (3.3%), intrathoracic lymph nodes (2.4%), osteoarticular (1%), and spine (0.9%). Tuberculous peritonitis is also rare in Germany. The diagnosis of any extrapulmonary forms of tuberculosis is quite difficult; in the case of peritoneal tuberculosis this is because clinical manifestations are non-specific, such as weight loss, abdominal pain, fever, ascites, vomiting [1-3]. The diagnosis of tuberculous peritonitis is often delayed on account of non-specific clinical signs or symptoms, absence of specific biological markers, long incubation times for cultures and non-specific radiographic or ultrasonographic signs. The prognosis in tuberculous peritonitis was unfavorable before treatment with antituberculous drugs became available and the mortality averaged 50 per cent [4].

## Case report

Two months before the patient visited our hospital she had been admitted to the emergency unit of another hospital with vomiting, abdominal pain and weight loss of 10 kg within three months. A diagnosis of spontaneous bacterial peritonitis was ruled out. Her clinical signs were initially attributed to severe gastritis and an ulcer in the pyloric canal. She had suffered from primary biliary cirrhosis (PBC) since 1992 and had been treated with 750 mg of ursodeoxycholic acid daily without immunosuppressive therapy. She had no significant past history of pulmonary or genital tuberculosis. She had given birth to a son and a daughter.

Physical examination showed a blood pressure of 120/60 mmHg; regular pulse at 84/min; and a body temperature of 39.6°C. Superficial lymph nodes were not palpable. Chest examination revealed basal breathing. The patient's abdomen was distended, and peristaltic sounds were not audible. Edema of the extremities was present. The initial laboratory data for blood (Table 1) rendered a high C-reactive protein (CRP) level of 15.68 mg/dl. Her tuberculin test was negative.

**Table 1:** Laboratory data for blood on admission

WBC	5.2/nl
Seg	81 %
Lymp	8 %
Mono	8 %
Eos	0 %
Baso	2 %
RBC	3.64/Pl
Hb	11.3 g/dl
Ht	34.2 %
Platelets	52/nl
Total protein	8.4 g/dl
Albumin	1.7 g/dl
GOT	41 U/l
GPT	16 U/l
LDH	301 U/l
ALP	217 U/l
Gamma-GT	90 U/l
Total bilirubin	1.7 ml/dl
CHE	2057 U/l
CRP	15.68 mg/dl
Creatinine	0.67 mg/dl
Na	135 mmol/l
K	4. 3 mmol/l
Lactate	2.47 mmol/l
AFP	1.7 ng/ml
CA 125	67.4 U/ml
CA 19-9	33 U/ml
CEA	3 ng/ml
Quick	45 %
PTT	45 Sec.

**Figure 1:**
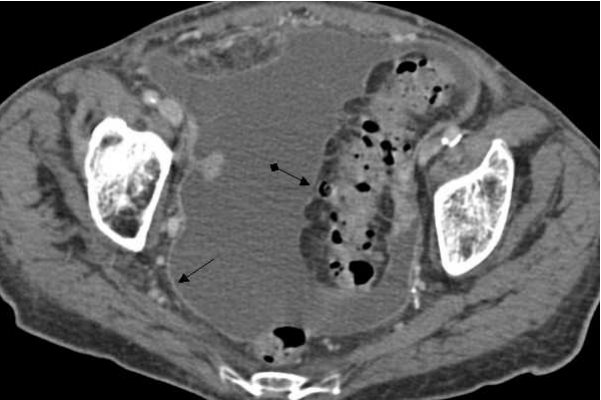
CT pelvis pronounced contrast enhancement of the peritoneum (); thickened wall of the sigmoid colon ().

CT scan of the chest showed bilateral pleural effusions without lymph node swellings. Abdominal ultrasonography revealed massive ascites with multiple septa. A CT scan of the abdomen showed a thickened intestinal wall located in the sigmoid colon (Fig. 1) and pronounced enhancement of the peritoneum. There were no masses or lymph node swellings in the abdominal cavity. Esophagogastroscopy and ileocoloscopy revealed no ulcer or stenosis in the colon or ileum.

The nature of ascites was revealed by puncture and findings are listed in Table 2, including a protein level of 4.6 g/dl. Microscopy was requested for malignant cells and *Mycobacterium*, neither of which was discovered. The cultures and polymerase chain reaction (PCR) analysis of stool and urine as well as from bronchial lavage were negative for *M. tuberculosis*, but the culture of ascites returned positive for *M. tuberculosis *after three weeks. The final diagnosis was tuberculous peritonitis.

**Table 2:** Laboratory data of ascites on admission

Protein	4.6 mg/dl
Glucose	60 mg/dl
LDH	442 U/l
Cholesterol	43 mg/dl
Leukocytes	0.3/nl

We began anti-tuberculous therapy using isoniazid, rifampicin, ethambutol, and pyrazinamide.

In the end, the fulminating course of the disease could not be positively influenced by this therapy and multi-organ failure with liver failure and nephritic failure developed.

## Discussion

Tuberculous peritonitis is always secondary to other tuberculous lesions. Tuberculous peritonitis appears to be more common in females than in males. Tuberculosis in females commonly reaches the peritoneum through tubal infection and attacks the tubes during the sexually active period of life. It may be due to either a local extension from a tuberculous lymph node, Fallopian tube, tuberculous intestinal ulcer, or may be caused by hematogenous or lymphatic spread from distant sources of infection [4]. Although this patient had no previous medical history of pulmonary or extra-pulmonary tuberculosis, and the CT scan of the chest and abdomen showed no lymph node swellings anywhere, we are certain that the tuberculous infection was based on reactivation of a long-latent tuberculous focus in the peritoneum due to her immunocompromised state following a prolonged course of primary biliary cirrhosis over 14 years.

On the basis of the history, examination and laboratory findings a differential diagnosis of spontaneous bacterial peritonitis, bacterial cholangitis, intra-abdominal malignancy or abdominal tuberculosis was considered. The patient was initially treated with high-dose broad-spectrum antibiotics. The patient's condition nevertheless continued to deteriorate and in addition to the CT and ultrasonographic findings, we had planned to perform a laparoscopy. During preparation for laparoscopy the culture of ascites returned positive for *M. tuberculosis *after three weeks.

Extrapulmonary manifestation of tuberculosis can be found in about 20.4 % of cases in German population [5]. The incidence of tuberculous peritonitis in Germany has been very low and tuberculosis of the intestinal tract was found in approximately 0.8% of tuberculosis cases in 2004 [5]. The 'golden rule' for a rapid diagnosis of tuberculous peritonitis is a laparoscopy-guided biopsy. But because of the anaesthetic and bleeding risk, laparoscopy-guided biopsy was not an immediately available option for our patient.

Positive cultures for *M. tuberculosis *have been reported from 7.8% in a small case report [6], up to 83% [7], which may be dependent on the fluid quantity. 1L of fluid was recommended by Singh *et al*. [7].

The use of PCR to detect *M. tuberculosis *was diagnostically useful in patients with ascites who were suspected of having tuberculous peritonitis in order to achieve a prompter diagnosis and treatment. The IS6110 primer was detected in 60% of specimens [8,9]. Unfortunately the PCR analysis of ascitic fluid was not performed in this case.

Adenosine deaminase (ADA) levels are used for diagnosing tuberculosis in several locations and have also been recommended in suspected tuberculous peritonitis. The pertinent literature judges the usefulness of ADA levels in ascitic fluid as a diagnostic test for peritoneal tuberculosis differently. Riquelme *et al*. reported that ADA levels showed a high sensitivity (100%) and specificity (97%) in ascitic fluid using Giusti's methods [1]. Marinez-Vazquez reported that ADA is not specific for tuberculous peritonitis [10]. Lower sensitivities were reported in the context of underlying liver cirrhosis, and false positives occurred in malignancy and bacterial peritonitis [10,11]. ADA levels were not measured in this instance.

The question, why the tuberculin test was negative in this case, cannot be answered easily. New types of immunological test methods such as the Quanti FERON – TB Gold in – tube (ELISA assay) and the T – SPOT – TB test (ELISPOT assay), which are based on the interferon γ (IFN – γ) production of sensitized T lymphocytes, may yet provide a useful additional diagnostic method. In patients with extrapulmonary tuberculosis, a sensitivity of the IFN – γ test of 92 % was observed, although only 13 patients were included in the study [12]. Unfortunately, these methods were not available in Germany at the time the patient was admitted.

In this case, massive ascites was observed with multiple fine delicate septa on ultrasonography, and a thickened intestinal wall located in the sigmoid colon and pronounced enhancement of peritoneum was seen on CT scan. Case reports [13] and small case studies in the literature have already reported these findings retrospectively and prospectively [14-17].

Although tuberculous peritonitis may be associated with alcoholic cirrhosis of the liver, patients with PBC usually have ascites, making the diagnosis more difficult. At the time of diagnosis the decision to initiate anti-tuberculous therapy turned out to be difficult due to concomitant serious liver failure and no histological or bacteriological confirmation of infection with *M. Tuberculosis*. Five days after the therapy commenced the patient died of liver and multiple organ failure. In hindsight, an anti-tuberculous treatment should have been started without waiting for the culture report.

## Conclusion

Tuberculous peritonitis must be considered in the initial differential diagnosis of patients with non-specific clinical signs and symptoms such as vomiting, abdominal pain, ascites, weight loss and fever that mimic the picture of spontaneous bacterial peritonitis in patients with PBC. The sonographic findings are not specific in tuberculous peritonitis, but can be useful in differentiating tuberculous ascites. An awareness of the ultrasonographic features may contribute valuable information, help in the diagnosis of tuberculous peritonitis, improve diagnostic accuracy and avoid clinical mismanagement.

## Abbreviations

ADA = adenosine deaminase activity; CT = computed tomography; IFN – γ = Interferon γ; M = Mycobacterium; PBC = primary biliary cirrhosis; polymerase chain reaction = PCR.

## Competing interests

The author(s) declare that they have no competing interests.

## Authors' contributions

YV was responsible for the patient's management; and manuscript design and drafting.

JB assisted with the manuscript draft and figures and provided general technical support.

GW was responsible for the radiological findings and provided the figures.

BH was responsible for the design, coordination and supervision of the patient's management.

All authors read and approved the final manuscript.

## Consent

Written informed consent was obtained from the patient's relatives for the publication of the study.
